# Developmental changes in action-outcome regularity perceptual sensitivity and its relationship to hand motor function in 5–16-year-old children

**DOI:** 10.1038/s41598-022-21827-8

**Published:** 2022-10-20

**Authors:** Satoshi Nobusako, Wen Wen, Yusuke Nagakura, Mitsuyo Tatsumi, Shin Kataoka, Taeko Tsujimoto, Ayami Sakai, Teruyuki Yokomoto, Emiko Takata, Emi Furukawa, Daiki Asano, Michihiro Osumi, Akio Nakai, Shu Morioka

**Affiliations:** 1 Neurorehabilitation Research Center, Kio University, 4-2-2 Umaminaka, Koryo-cho, Kitakatsuragi-gun, Nara, 635-0832 Japan; 2 Graduate School of Health Science, Kio University, 4-2-2 Umaminaka, Koryo-cho, Kitakatsuragi-gun, Nara, 635-0832 Japan; 3 Research Into Artifacts, Center for Engineering, and Department of Precision Engineering, The University of Tokyo, 7-3-1 Hongo, Bunkyo-ku, Tokyo, 113-8656 Japan; 4 Department of Orthopedics and Rehabilitation, Tatsue Clinic, 2-2-2 Kigawahigashi, Yodogawa-ku, Osaka-city, Osaka 532-0012 Japan; 5 Sawayaka Dental Clinic, 191-14 Nakasozhi-cho, Kashihara, Nara 634-0845 Japan; 6 Department of Rehabilitation, Nishide Clinic, 6-10-1 Higashi-Mikuni, Osaka-city, Osaka 532-0002 Japan; 7 Department of Rehabilitation, Higashi Osaka Yamaji Hospital, 1-7-5 Inaba, Higashi Osaka-city, Osaka, 578-0925 Japan; 8 Faculty of Education, Kio University, 4-2-2 Umaminaka, Koryo-cho, Kitakatsuragi-gun, Nara, 635-0832 Japan; 9 Department of Rehabilitation, Japan Baptist Hospital, 47 Yamanomoto-cho, Sakyouku, Kyoto-city, Kyoto 606-8273 Japan; 10 Graduate School of Clinical Education & The Center for the Study of Child Development, Institute for Education, Mukogawa Women’s University, 6-46 Ikebiraki-cho, Nishinomiya-city, Hyogo 663-8558 Japan

**Keywords:** Neuroscience, Psychology

## Abstract

Along with the comparator model, the perception of action-outcome regularity is involved in the generation of sense of agency. In addition, the perception of action-outcome regularity is related to motor performance. However, no studies have examined the developmental changes in the perception of action-outcome regularity. The current study measured perceptual sensitivity to action-outcome regularity and manual dexterity in 200 children aged between 5 and 16 years. The results showed that perceptual sensitivity to action-outcome regularity was significantly lower in 5–6-year-old children than in 9–16-year-old children, and that it was significantly lower in children with low manual dexterity than in children with medium to high manual dexterity. Correlation analyses revealed significant correlations of age and perceptual sensitivity to action-outcome regularity, but no significant correlation of manual dexterity and perceptual sensitivity to action-outcome regularity, either overall or in any age band. The present study suggests that perceptual sensitivity to action-outcome regularity is immature at 5–6 years of age and that it may be impaired in 5–16-year-old children with poor manual dexterity.

## Introduction

The comparator model in central monitoring theory plays an important role in motor control and motor learning^1,2^. Before slow sensory-motor feedback becomes available, the comparator model provides stability to the motor system by predicting the outcome of movements, which allows for rapid online corrections^3,4^. During the production of a motor plan, the motor command is generated by the motor cortex and relayed to the body. An efference copy of this command, i.e., predicted sensory feedback, is compared to the actual sensory feedback. The discrepancy between the motor prediction and actual sensory feedback, i.e., prediction error, allows for rapid online modification of the motor command. From a developmental perspective, sensory-motor functions in the comparator model undergo developmental changes from school age to adolescence and the performance of the comparator model improves with age^5–10^. For example, performance in the double-step reaching task, which reflects online motor control ability, improves between 6–7-, 8–12-, and 13–17-year-old children^5^.

The comparator model is also involved in generating the subjective feeling of controlling external events, i.e., “sense of agency” (SoA)^11,12^. That is, the results of movements are experienced as being self-caused if the predicted sensory feedback matches the actual sensory feedback; however, in case of mismatch, they are experienced as being generated externally^13^. In fact, SoA typically decreases with increasing temporal or spatial discrepancies between self-generated movements and their sensory feedback^14–23^. In recent years, there has been increasing interest in whether SoA changes with development or remains constant throughout life. Metcalfe et al.^24^ and van Elk et al.^25^ showed that explicit SoA is similar in school-age children and young adults, and that SoA is increased in children when the outcome is positive, even in the presence of prediction error. This tendency is even stronger in preschoolers^26^. Aytemur & Levita^27^ demonstrated that the intentional binding effect, a measure of implicit SoA, increases similarly in childhood and adulthood, decreases from mid-adolescence, and reaches a minimum in late adolescence. Blakey et al.^28^ reported that the intentional binding effect is present at 4 years of age and does not change until 11 years of age, and Lorimer et al.^29^ showed that the intentional binding effect does not change from 6 to 10 years of age and its strength is similar to that of adults. Conversely, Cavazzana et al.^30^ suggested that the intentional binding effect is smaller in children than in adults. According to the comparator model, SoA is expected to increase with age from childhood to adolescence^5–10^, but a summary of previous studies examining developmental changes in SoA indicates that it tends to decrease from childhood to adolescence^24–29^. Therefore, in the current study, we focused on another important source of SoA information other than the comparator model, i.e., the perception of action-outcome regularity^31–34^, and examined whether developmental changes in perceptual sensitivity to action-outcome regularity could be one of the reasons for this discrepancy. Recently, the perception of action-outcome regularity was reported to be involved in generating subjective SoA, which was maintained when the regular action-outcome relationship was maintained, even when the prediction error of the comparator model was large^31^. In adults, when the percentage of self-generated movements is > 50%, the perceptual accuracy of action-outcome regularity improves and SoA occurs^31,32^.

Importantly, action-outcome regularity detection differs from comparator model-based processes of motor control in that the former does not require a precise prediction of the outcome of each action. Developmental research offers some support for the idea of a regularity detection mechanism. Infants aged 9–12 weeks make more foot thrust movements when their ankle is attached to an overhead suspension bar, so that their movements produce visual effects^35^. In addition, the sucking behavior of 2–4-month-old infants is enhanced by visual and auditory feedback^36,37^. Infants at this age have only minimal motor skills and lack the precise forward and inverse models required for targeted movements. Therefore, the reinforcement of their exploratory behavior is probably due to the perception of a regular relationship between events in the external world and their own actions. However, it is unknown whether the perceptual sensitivity of action-outcome regularity undergoes developmental changes from childhood to adolescence. Since previous studies have shown that SoA increases when the outcome is positive, especially in preschoolers^24–26^, the present study examined the developmental changes in perceptual sensitivity to action-outcome regularity involved in the generation of SoA in 4–16-year-old children.

A study examining the relationships between age, manual dexterity, and the ability to detect action-outcome temporal errors in 4–15-year-old children showed that age and manual dexterity are significant independent predictors of comparator model function^8^. Significant correlations were also found between improved comparator model function and improved manual dexterity in 4–15-year-old children^9^ and between a shorter time window of SoA, i.e., the period in which the temporal error between action and outcome maintains SoA, and higher manual dexterity in school-age children, but not in adults^21^. Similar results have been observed in typically developing 8–11-year-old children^22^. Thus, there appears to be a relationship between the function of the comparator model involved in the generation of SoA and manual dexterity in children. As such, we also examined the relationship between manual dexterity and perceptual sensitivity to action-outcome regularity in the generation of SoA as well as the comparator model.

## Materials and methods

### Participants

A total of 225 children with typical development aged 4–16 years who were enrolled in regular classes at public preschools, primary schools, and secondary schools in Osaka and Nara, Japan, were recruited; however, 25 were excluded since they were unable to complete the experimental tasks. The remaining 200 children (mean age ± standard deviation, 9.31 ± 2.55 years; range, 5–16 years; 79 male; 187 right-handed) completed the experimental tasks. The dominant hand was self-reported as the preferred hand (right or left). Tables 1, 2 and 3 show the age, sex, and preferred hand of the 200 children. Information on the 25 individuals who could not complete the task is as follows: 4-year-old, *n* = 12, 8 male, 11 right-handed; 5-year-old, *n* = 6, 2 male, 5 right-handed; 6-year-old, *n* = 2, 1 male, 2 right-handed; 7-year-old, *n* = 1, female, right-handed; 8-year-old, *n* = 2, 2 female, 2 right-handed; 10-year-old, *n* = 1, female, right-handed; and 11-year-old, *n* = 1, female, right-handed. Of these, 24 children, excluding a 5-year-old right-handed boy who was unable to complete the manual dexterity test, were unable to complete the action-outcome regularity detection task. None of the 4-year-old children (*n* = 12) could complete the action-outcome regularity detection task.

**Table 1:** Summary of data for all participating children.

*n* = 200	Age (years)	Sex	Preferred hand	Manual dexterity		RDT
				Standard score	Percentile score	
Mean	9.31	Male = 79 Female = 121	Right = 187 Left = 13	12.21	70.5	0.581
Standard deviation	2.55			2.95	25.9	0.437
Range	5–16			4–18	2–99.5	0.169–3.171
Skewness	0.311			− 0.303	− 0.909	3.232
Kurtosis	− 0.596			− 0.036	− 0.046	12.002

*RDT* action-outcome regularity detection threshold.

**Table 2:** Summary of data for each age group.

Group		Age (years)	Sex	Preferred hand	Manual dexterity		RDT
					Standard score	Percentile	
5–6-year-old	Mean	5.74	Male = 19 Female = 12	Right = 30 Left = 1	12.10	69.5	1.017
	SD	0.44			3.08	27.8	0.735
7-year-old	Mean	7.00	Male = 14 Female = 14	Right = 27 Left = 1	12.96	77.1	0.504
	SD	0.00			2.67	20.1	0.112
8-year-old	Mean	8.00	Male = 8 Female = 15	Right = 21 Left = 2	12.35	71.5	0.528
	SD	0.00			3.04	27.5	0.292
9-year-old	Mean	9.00	Male = 10 Female = 16	Right = 26 Left = 0	12.81	75.7	0.458
	SD	0.00			2.56	19.7	0.141
10-year-old	Mean	10.00	Male = 5 Female = 19	Right = 21 Left = 3	13.46	83.0	0.443
	SD	0.00			2.02	15.8	0.106
11-year-old	Mean	11.00	Male = 11 Female = 17	Right = 26 Left = 2	10.71	56.5	0.595
	SD	0.00			2.98	27.2	0.504
12–16-year-old	Mean	13.05	Male = 12 Female = 28	Right = 36 Left = 4	11.60	65.1	0.481
	SD	1.18			3.24	30.0	0.343

*RDT* action-outcome regularity detection threshold, *SD* standard deviation.

**Table 3:** Summary of data for each manual dexterity group.

Group		Age (years)	Sex	Preferred hand	Manual dexterity		RDT
					Standard score	Percentile	
Low	Mean	10.35	Male = 9 Female = 8	Right = 16 Left = 1	6.29	12.6	1.195
	SD	3.57			1.26	7.2	0.893
Medium–low	Mean	9.62	Male = 17 Female = 20	Right = 35 Left = 2	9.54	44.0	0.469
	SD	2.30			0.51	6.6	0.295
Medium–high	Mean	9.11	Male = 24 Female = 30	Right = 49 Left = 5	11.57	69.9	0.542
	SD	2.62			0.50	6.0	0.350
High	Mean	9.11	Male = 29 Female = 63	Right = 87 Left = 5	14.75	92.2	0.536
	SD	2.37			1.51	5.6	0.305

*RDT* action-outcome regularity detection threshold, *SD* standard deviation.

The exclusion criteria were: (1) a general medical condition, e.g., cerebral palsy, hemiplegia, and muscular dystrophy; (2) diagnosis of a developmental disorder, e.g., autism spectrum disorder, attention deficit hyperactivity disorder, developmental coordination disorder, and learning disorder; or (3) diagnosis of intellectual disability. Eligibility was confirmed by interviewing parents and the results of regular checkups, which were provided by the doctor at each school. All experimental procedures were approved by the local ethics committee of the Graduate School and Faculty of Health Sciences at Kio University (approval number: R1-22). There were no foreseeable risks, and no personally identifying information was collected. The participants and their parents provided background information and written informed consent. The procedures complied with the ethical standards of the 1964 Declaration of Helsinki regarding the treatment of human participants in research.

### Procedures

The participants completed two experimental tasks: the action (motor)-outcome (visual) regularity detection task and the manual dexterity test of the Movement Assessment Battery for Children-2nd edition (M-ABC2). As the order in which the participants performed the tasks was not arranged by age, both tests were administered to each participant in a random order. The time required to complete each test was less than 20 min; both tests were completed within 40 min by each participant.

### Action-outcome regularity detection task

**Figure 1:**
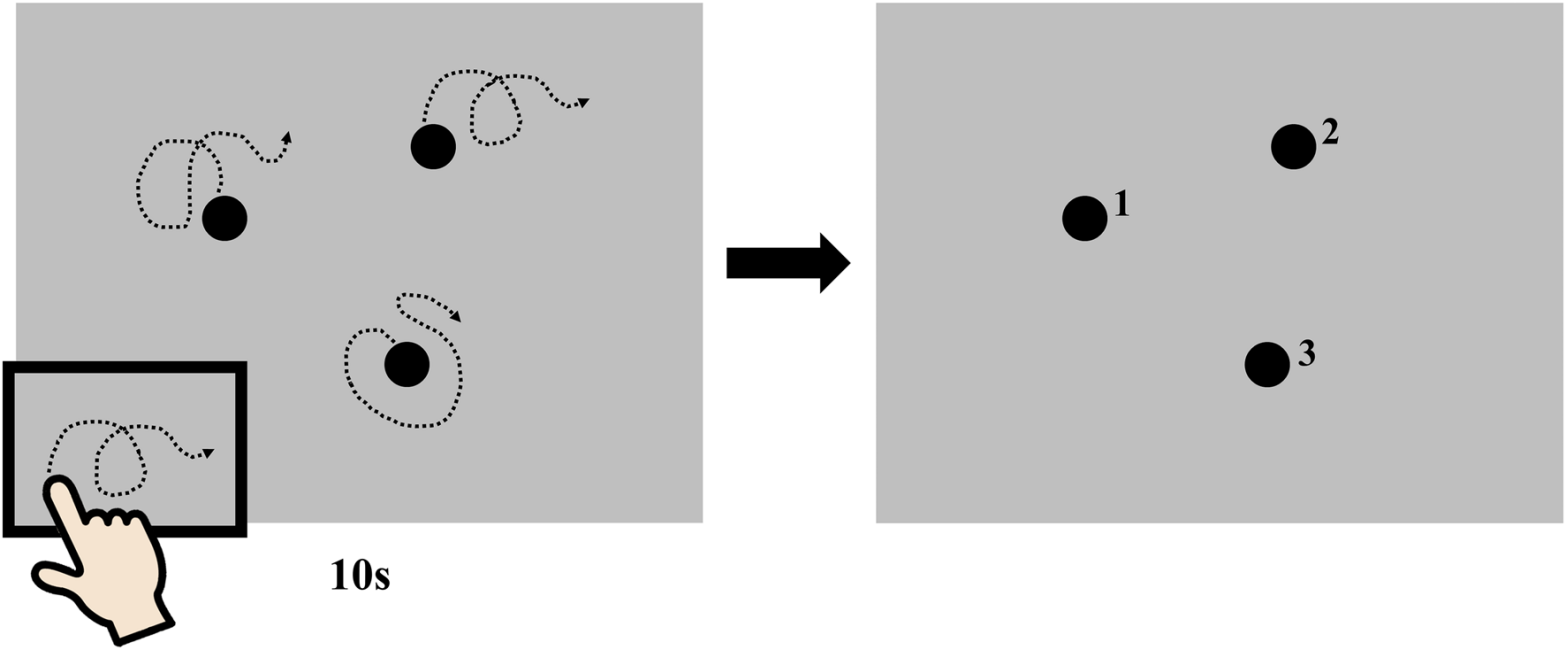
Action-outcome regularity detection task. The participant was allowed to move their finger freely on a touchpad to elicit motion of three dots for 10 s, and then stated which dot they thought they could control, i.e., the movement of which dot corresponded to their own finger movements. Two dots moved according to the pre-recorded movements of one of the authors, and one target dot moved in a hybrid direction that mixed the motion of the participant’s finger and the pre-recorded movements in a ratio of 0:100; 20:80; 40:60; 50:50; 60:40; 80:20; or 100:0. The dashed curves with arrows show the trajectories of the dots and the movements of the participant’s finger on the touchpad.

The participants were tested individually in a quiet testing room. The experimental task was the same as described previously^31^. The task was conducted using a computer, 17-inch LCD monitor (width 338 × height 270 mm, resolution 1280 × 1024 pixels at 60 Hz), keyboard, and touchpad (vertical 53 mm × width 101 mm). In the action-outcome regularity detection task (Fig. 1), the participant pressed the space bar on the keyboard to start each trial, and then saw three 10.5-mm (40 pixels) black dots on a light-gray screen. The initial positions of the dots were generated randomly, ensuring a minimal distance of 52.8 mm (200 pixels) between the dots and a maximal distance of 66 mm (250 pixels) from the center of the screen. Once the participant started to move their index finger of the preferred hand on the touchpad, all three dots started to move. The onset, offset, and velocity of dot motion corresponded to the movements of the participant’s finger on the touchpad, but the relationship between the dots’ trajectories and the participant’s finger movements varied. The motion of the target dot was a combination of the participant’s finger movements and randomly selected sections from 10,000 pre-recorded continuous finger movements generated by one of the authors. For the pre-recorded movements, the shift in the *x*- and *y*-axes of the finger position at each frame compared to the previous frame was recorded at a frequency of 60 Hz; the author was instructed to make the movements as diverse as possible, to cover all possible directions. The pre-recorded movements were combined with the participant’s real-time movements in each frame according to a certain ratio depending on the level of control^38^. The algorithm for combining the pre-recorded movements with the participant’s movements was as reported previously^38^. Specifically, in each frame when the screen was refreshed at a rate of 60 Hz, two moving angles were calculated from the participant’s real-time movement (α) and pre-recorded movement (β), respectively. A section of the pre-recorded movements was used frame by frame to ensure that they were smooth and continuous. Thereafter, the two movement angles were combined according to their weighting based on the control level to generate the movement direction of the dot on the screen. For example, in the 70% control condition, the generated movement angle was 0.7α + 0.3β. Finally, the magnitude of dot movement was equalized to the magnitude of the participant’s finger movement. Among the three dots, the detection target dot’s movements were 0%, 20%, 40%, 50%, 60%, 80%, and 100% control; the other two dots were 0% control. That is, the movement of the target dot was related to the participant’s finger movements, but distorted by a mixture of the pre-recorded movements, while the other two dots always moved on the trajectories of the pre-recorded movements. In each trial, the participant moved the dots freely for 10 s. Then, the three dots stopped moving and a number (1, 2, or 3) appeared near each of them. The experimenter first asked the question "Which dot did you feel you were controlling?" followed immediately by "Which dot did you feel reflected your finger movements?” These two questions were designed to be complementary to each other and to prevent a lack of understanding caused by the use of only one question. All participants were asked both questions in the same order. The participant pressed a number key (1, 2, or 3) to respond; if they could not respond with a number, they responded by pointing. Each participant performed six practice trials with the condition of 60%, 80%, and 100% control, each repeated twice. In the actual task, each condition was repeated six times at random, and 42 trials were completed.

### Action-outcome regularity detection sensitivity analysis

For each participant, the action-outcome regularity detection probability for each control condition was calculated. Logistic curves were fitted to the action-outcome regularity detection probability using the following formula^39,40^:

$$P(t)= \frac{1}{1+\mathrm{exp}(-a(t-{t}_{PSE}))}$$

where *t* is the control level, i.e., the ratio of the participant’s finger movements to the target dot movements; *P(t)* is the action-outcome regularity detection probability; *a* indicates the steepness of the fitted curve; and *tPSE* indicates the control level under which the probability of detecting the action-outcome regularity is 50%. The analyses were carried out with MATLAB R2016a (MathWorks, Natick, MA, USA) using the generalized linear model function in the Statistics and Machine Learning Toolbox.

The chance level of the regularity detection task was 33.3% because the participants chose one target among three dots. Therefore, we chose 66.6% as the perceptual threshold of regularity, because this value is the median between the chance level and maximum value. The regularity detection threshold (RDT), at which the probability of regularity detection is 66.6%, was calculated based on the following equation:

$$x=PSE-( \frac{1}{a} )\times log(\frac{1-y}{y}, 2.7182818)$$

where *PSE* is the control level at which the regularity detection probability is 50%; *a* indicates the steepness of the fitted curve; *y* indicates the regularity detection probability of 66.6%; and *x* indicates the control level that is the action-outcome RDT for each participant. The RDT served as a quantitative measure of sensitivity to action-outcome regularity detection for each participant. A decrease in the RDT indicated a higher sensitivity to detect action-outcome regularity and vice versa.

### Manual dexterity test of the M-ABC2

The manual dexterity test of the M-ABC2^41^ is a standardized, age-adjusted test to identify motor problems in children, in which different tasks are administered according to age. The M-ABC2 has good test–retest reliability with a minimum value at any age of 0.75, inter-rater value of 0.70, and concurrent validity^41^. This test has three age bands: 3–6, 7–10, and 11–16 years.

The 5–6-year-old children were administered the posting coins, threading beads, and drawing trail I tests. The 7–10-year-old children were administered the placing pegs, threading lace, and drawing trail II tests. The 11–16-year-old children were administered the turning pegs, triangle with nuts and bolts, and drawing trail III tests. According to the manual of the M-ABC2, the standard and percentile scores of the participants were calculated from the raw scores. The standard and percentile scores reflect the degree of manual dexterity for each year of age, in which higher scores represent an improvement of manual dexterity within each age group. A specifically trained and certified physical therapist administered all of these assessments.

### Statistical analysis

The purpose of the current study was to determine if there are developmental changes in the detection sensitivity of action-outcome regularity and their relationship to manual dexterity. Therefore, the data were divided according to age band and manual dexterity. The age groups were 5–6 (*n* = 31), 7 (*n* = 28), 8 (*n* = 23), 9 (*n* = 26), 10 (*n* = 24), 11 (*n* = 28), and 12–16 (*n* = 40) years. The manual dexterity groups were low (percentile score range, 0.1–25; standard score range, 1–8; *n* = 17), medium–low (percentile score range, 26–50; standard score range, 9–10; *n* = 37), medium–high (percentile score range, 51–75; standard score range, 11–12; *n* = 54), and high (percentile score range, 76–99.9; standard score range, 13–19; *n* = 92) according to the results of the manual dexterity test.

The chi-square test for independence was used to compare sex and preferred hand for each age group. Since manual dexterity (standard score) in each age group was normally distributed according to the Shapiro–Wilk test, one-way analysis of variance was used for comparisons; Bonferroni’s method was used for multiple comparisons in *post-hoc* analysis. Since the RDT in each age group was not normally distributed according to the Shapiro–Wilk test, it was compared using the Kruskal–Wallis test; the Mann–Whitney U test was used for *post-hoc* analyses.

The chi-square test for independence was used to compare sex and preferred hand for each manual dexterity group. Age and the RDT in each manual dexterity group were compared using the Kruskal–Wallis test, because they were not normally distributed according to the Shapiro–Wilk test; the Mann–Whitney U test was used for multiple comparisons in *post-hoc* analysis.

Since the overall (*n* = 200) age, manual dexterity, and RDT were not normally distributed according to the Shapiro–Wilk test, correlation analysis was performed using Spearman’s rank correlation coefficient. In addition, correlation analysis was performed between manual dexterity and the RDT in each age group. The Shapiro–Wilk test showed that manual dexterity and the RDT were normally distributed in the 7- and 9-year-old groups, but not in the other age groups. Therefore, Pearson’s product-moment correlation coefficient was used to analyze the correlation between manual dexterity and the RDT in the 7- and 9-year-old groups, and Spearman’s rank correlation coefficient was used to analyze the correlation in the other age groups.

The significance level was set at α = 0.05 for all analyses, and Bonferroni’s correction was used to adjust for multiple comparisons in *post-hoc* analyses. In addition, the effect size (*r*, *d*, *η*^2^, *ω*^2^) was also calculated. In correlation analysis, Bonferroni’s correction of the *p*-value and the false discovery rate (*q*-value = 0.05) using the Benjamini and Hochberg method was performed^42^. All statistical analyses were performed using SPSS ver. 26 (IBM Corporation, Armonk, NY, USA).

### Ethics statement

The experimental procedures were approved by the local ethics committee of the Faculty of Health Sciences at Kio University (approval number: R1-22). Therewere no foreseeable risks to the participants; no personally identifying information was collected. The children and their parents provided background information and written informed consent. The procedures complied with the ethical standards of the 1964Declaration of Helsinki regarding the treatment of human participants in research.

## Results

Table 1 shows a summary of the age, sex, preferred hand, manual dexterity, and RDT for all participating children. The mean ± standard deviation of the RDT was 0.581 ± 0.437, indicating that action-outcome regularity could be detected when the level of control exceeded 50%.

**Figure 2:**
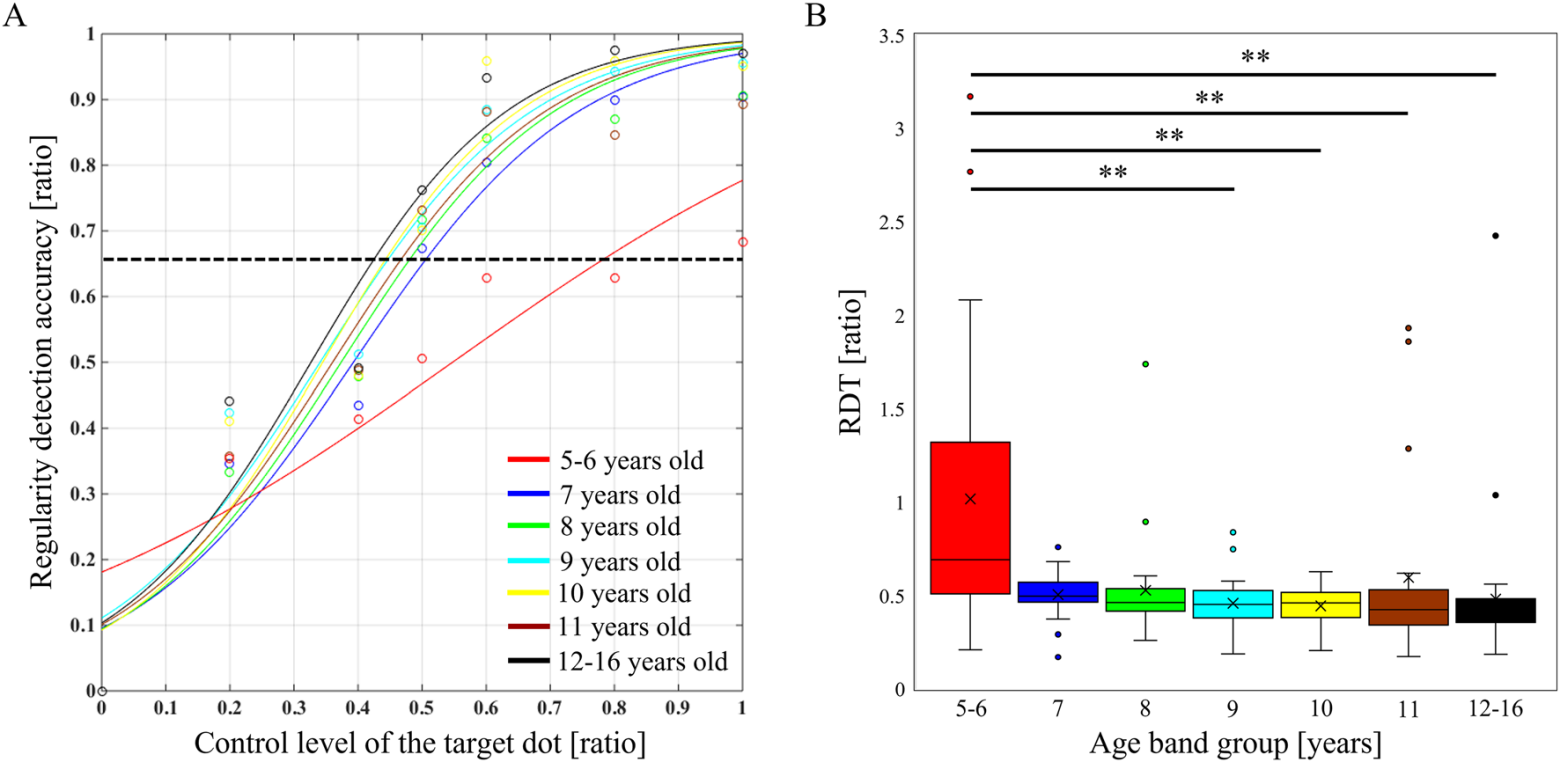
Detection probability curves of action-outcome regularity in each age group and their comparison. (**A**) The colored curves show the detection probability curves of action-outcome regularity for each age group. The black dashed line represents the action-outcome regularity detection threshold (66.6%). (**B**) Comparative results of the regularity detection threshold (RDT) for each age group. Lines represent the range of the minimum and maximum. Boxes represent the lower, median, and upper quartiles. *p < 0.05, **p < 0.01.

Table 2 shows a summary of each age group. The chi-square test for sex comparisons for each age group showed a significant difference (*χ*^*2*^(6) = 12.688, *p* = 0.048, *φ* = 0.252). Residual analysis demonstrated that there were significantly fewer females among the 5–6-year-old children and significantly fewer males among the 10-year-old children. A chi-square test of the comparison of preferred hand for each age group revealed no significant difference (*χ*^*2*^(6) = 5.179, *p* = 0.521, *φ* = 0.161). There was a significant difference in the comparison of the manual dexterity of each age group (*F* (6, 199) = 2.855, *p* = 0.011, *η*^2^ = 0.08, *ω*^2^ = 0.05). *Post-hoc* analysis showed that manual dexterity was significantly higher in 10-year-old children than in 11-year-old children (*t* (50) = 3.819, *p* = 0.015, *r* = 0.48, *d* = 1.06), but there was no significant difference among the other age groups. The action-outcome regularity detection probability curves for each age group are shown in Fig. 2A, and the comparisons of the RDT for each age group are shown in Fig. 2B. The Kruskal–Wallis test revealed a main effect of the RDT between the age groups (*p* < 0.001). *Post-hoc* analysis showed that the RDT was significantly higher in 5–6-year-old children than in 9- (*z* = − 3.653, *p* = 0.006, *r* = − 0.484), 10- (*z* = − 3.674, *p* = 0.005, *r* = − 0.495), 11- (*z* = − 3.173, *p* = 0.002, *r* = − 0.413), and 12–16-year-old (*z* = − 4.475, *p* < 0.001, *r* = − 0.531) children. As 5–6-year-old children had significantly fewer females and 10-year-old children had significantly fewer males, additional statistical analyses included comparisons between males and females for manual dexterity and the RDT in 5–6- and 10-year-old children, respectively. However, there was no significant sex differences in manual dexterity and the RDT in the 5–6- (manual dexterity, *z* = − 0.371, *p* = 0.734, *r* = − 0.067; RDT, *z* = − 0.649, *p* = 0.535, *r* = − 0.117) and 10-year-old (manual dexterity, *z* = − 1.556, *p* = 0.139, *r* = − 0.318; RDT, *z* = − 0.107, *p* = 0.945, *r* = − 0.022) children.

**Figure 3:**
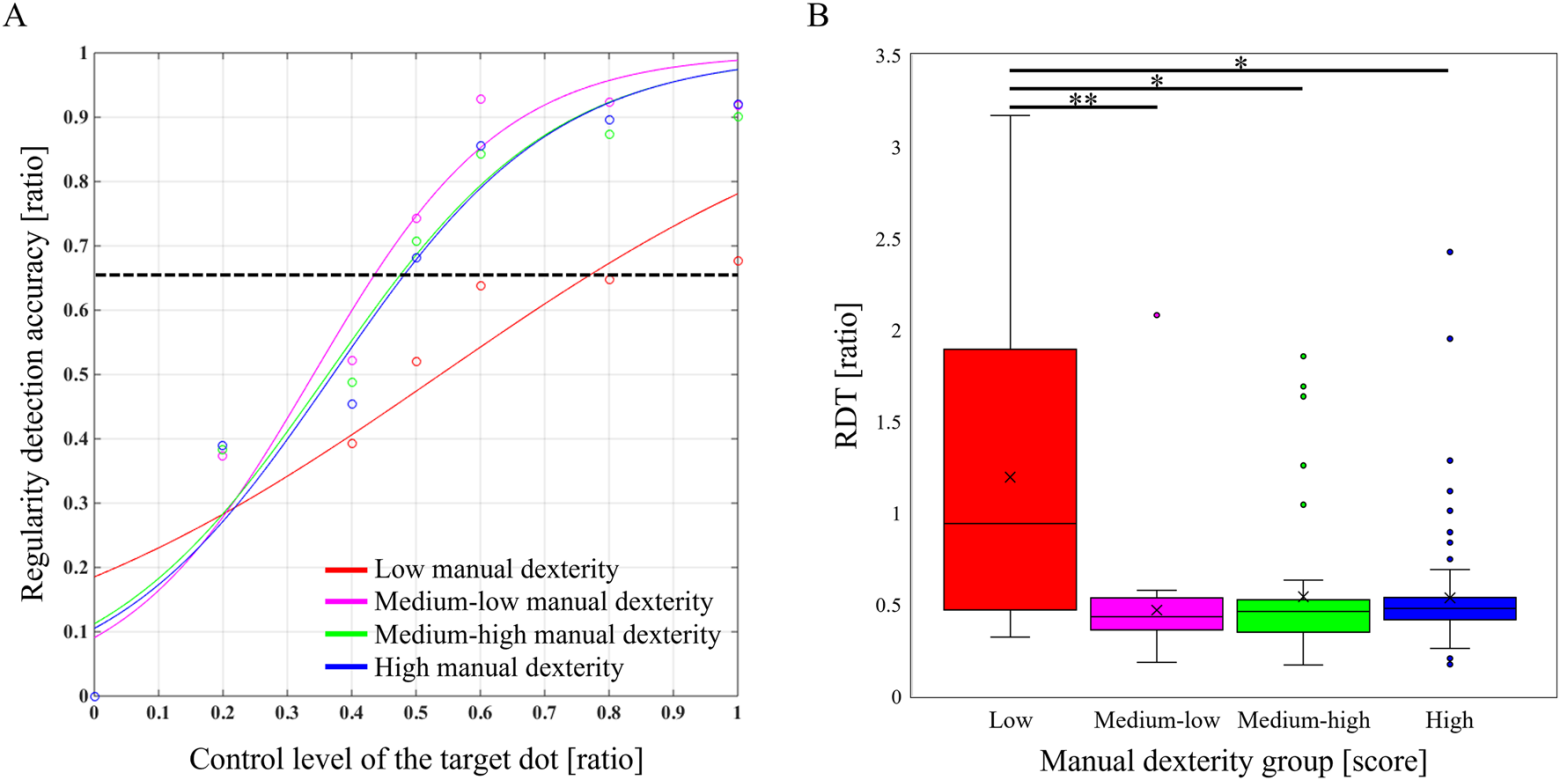
Detection probability curves of action-outcome regularity in each manual dexterity group and their comparison. (**A**) The colored curves show the detection probability curves of action-outcome regularity for each manual dexterity group. The black dashed line represents the action-outcome regularity detection threshold (66.6%). (**B**) Comparative results of the regularity detection threshold (RDT) for each manual dexterity group. Lines represent the range of the minimum and maximum. Boxes represent the lower, median, and upper quartiles. *p < 0.05, **p < 0.01.

Table 3 shows a summary of each manual dexterity group. Chi-square tests for sex and preferred hand for each manual dexterity group showed no significant differences (sex, *χ*^*2*^(3) = 4.931, *p* = 0.177, *φ* = 0.151; preferred hand, *χ*^*2*^(3) = 0.932, *p* = 0.818, *φ* = 0.068). There was no significant difference in age between each manual dexterity group (*p* = 0.314). The action-outcome regularity detection probability curves for each manual dexterity group are shown in Fig. 3A, and the comparisons of the RDT for each manual dexterity group are shown in Fig. 3B. The RDT was significantly different in each manual dexterity group (*p* = 0.004). *Post-hoc* analysis revealed that the RDT was significantly higher in the low manual dexterity group than in the medium–low (*z* = − 3.260, *p* = 0.002, *r* = − 0.444), medium–high (*z* = − 3.005, *p* = 0.011, *r* = − 0.357), and high (*z* = − 2.931, *p* = 0.035, *r* = − 0.281) manual dexterity groups.

**Figure 4:**
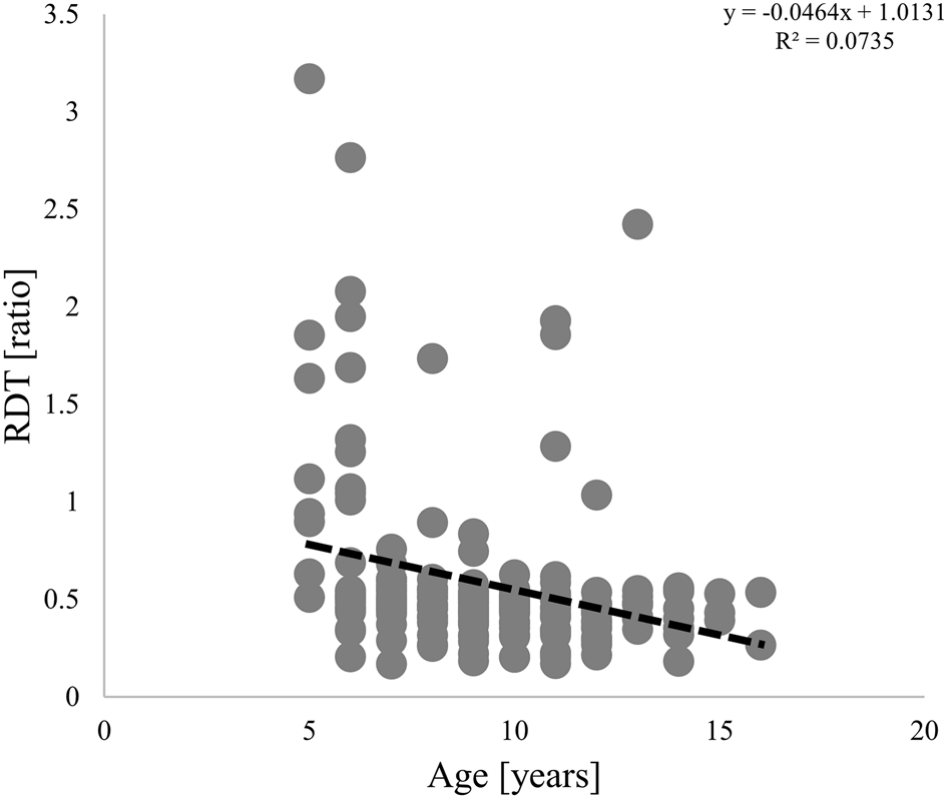
Scatter plot showing the relationship between age and the regularity detection threshold (RDT) for all participating children (*n* = 200).

Correlation analysis of age, manual dexterity, and the RDT for all participants showed a significant negative correlation between age and the RDT (*r* = − 0.358, *p* < 0.001, *q* < 0.001) (Fig. 4). There was no significant correlation between manual dexterity and age or the RDT (manual dexterity and age, *r* = − 0.125, *p* = 0.236, *q* = 0.118; manual dexterity and RDT, *r* = − 0.018, *p* = 2.406, *q* = 0.802).

In all age groups, there was no significant correlation between manual dexterity and the RDT (5–6-year-old, *rs* = − 0.403, *p* = 0.172, *q* = 0.172; 7-year-old, *r* = 0.104, *p* = 4.197, *q* = 1.049; 8-year-old, *rs* = − 0.031, *p* = 6.217, *q* = 1.036; 9-year-old, *r* = 0.041, *p* = 5.904, *q* = 1.181; 10-year-old, *rs* = 0.118, *p* = 4.076, *q* = 1.359; 11-year-old, *rs* = − 0.151, *p* = 3.108, *q* = 1.554; 12–16-year-old, *rs* = − 0.003, *p* = 6.897, *q* = 0.985).

## Discussion

In total, 225 children aged 4–16 years participated in this study, but none of the 4-year-old children (*n* = 12) were able to complete the action-outcome regularity detection task, indicating that this task was only applicable to children aged 5 years and older. Therefore, we examined the relationship between developmental changes in perceptual sensitivity to action-outcome regularity and its relationship with manual dexterity in 5–16-year-old children. The results showed that the RDT was significantly higher in 5–6-year-old children than in 9–16-year-old children. In overall correlation analysis, there was a significant negative correlation between age and the RDT. Our findings indicate that the age of 5–6 years is probably a critical period for the development of the ability to detect regular spatial transformation between one’s own actions and an external stimulus. Although there was no significant difference in age between groups based on manual dexterity, the RDT was significantly higher in children with low manual dexterity than in those with medium to high manual dexterity.

We found that the perception of action-outcome regularity was not as well developed in 5–6-year-old children as in 9–16-year-old children. The increase of perceptual sensitivity to action-outcome regularity with increasing age was evident in the significant negative correlation between age and the RDT. There was a significant difference in sex among each age group, with significantly fewer females in the 5–6-year-old children and significantly fewer males in the 10-year-old children. However, there was a significant difference in the RDT between the 5–6- and 9–16-year-old children. There was no significant sex difference in the RDT in the 5–6- or 10-year-old children. In addition, there was no significant difference in preferred hand among the age groups. Therefore, it is unlikely that the differences in sex and preferred hand between age groups had an effect on the significant difference in the RDT between age groups.

This may be due to a switch from local to global processing patterns during the development of information processing of visual stimuli. Normally, adults show a global processing pattern for visual stimuli, which is the ability to see and judge local and whole areas, but young children are more likely to show a local processing pattern. Four-year-old children exhibit a local bias toward visual stimuli, but gradually shift from local to global processing at 5–6 years of age, evolving to an adult-like global preference at 9 years of age^43–46^. Some studies have provided evidence that children aged up to 6 years have a local bias^46–48^. Thus, it is possible that the children under 6 years of age were hindered from selecting the self-controlled dot in comparison to the other dots in the current experimental task because they were unable to see and judge the whole area.

In addition, 5–6-year-old children have more difficulty in disengaging attention from competitive items, e.g., non-target distractors, and are less likely to focus on a target than 9-year-old children and adults^49,50^. The development of visual search skills, e.g., detecting a target among multiple visual stimuli, depends on the development of executive functions^51^, and it has been shown that executive attention, i.e., the capacity to ignore distractors, improves at 7–8 years of age, compared to 6 years and younger^52^. After children enter primary school, they become increasingly efficient at ignoring irrelevant stimuli^49^. Moreover, 5–7-year-old children have more difficulties with response inhibition than 9–11-year-old children, but they gain more efficient control over their behavior as they age, which drives developmental improvements in response inhibition^53^. Therefore, in the present study, the perception of action-outcome regularity was significantly lower in 5–6-year-old children than in 9–16-year-old children because they have a dominant local processing pattern for visual stimuli and their control of visual attention is immature.

Previous studies suggested that SoA is similar in childhood and adulthood^24,25,27,29^, but also that it decreases from childhood to adolescence^27^. Conversely, the comparator model involved in the generation of SoA has been shown to improve from childhood to adolescence^5–10^. One of the purposes of this study was to investigate whether developmental changes in perceptual sensitivity to action-outcome regularity could account for this discrepancy. However, the current results do not resolve this issue, but show that perceptual sensitivity to action-outcome regularity is still immature in 5–6-year-old children. Therefore, future studies should examine the relationship between the developmental changes in the perceptual sensitivity of action-outcome regularity and SoA using SoA tasks, such as the intentional binding task, in addition to the action-outcome regularity detection task used in the current study. For example, if the perceptual sensitivity of action-outcome regularity differs between 5–6- and 9–16-year-old children, as our results suggest, it may be possible to examine whether this makes a difference in the intentional binding effect. Such studies may not only examine developmental changes in SoA but may also lead to the identification of developmental changes in the background factors that generate SoA.

Several studies have shown significant correlations between the ability to detect action-outcome discrepancies and manual dexterity in 4–15-year-old children^8,9^, and between the time window of SoA and manual dexterity in 6–12-year-old children^21,22^. However, our results did not show a significant correlation between perceptual sensitivity of action-outcome regularity and manual dexterity. The experimental tasks used in these previous studies involved the insertion of a linear disturbance as a temporal error between an action and its outcome, and detected the discrepancies or answered whether or not SoA was present^8,9,21,22^. The comparator model has important roles in comparing an action with actual sensory feedback, generating error signals, and correcting the motor commands online^1–4^. Importantly, error signals also act as training signals to refine the accuracy of predictive models, and this iterative process is fundamental for motor learning^54^. Therefore, an increase in the ability to detect linear action-outcome disturbances may improve motor performance; conversely, a reduction in this ability may hinder the generation of error signals and impair motor performance. Therefore, the results of previous studies may have indicated a significant correlation of manual dexterity with the temporal error detection function and the time window of SoA^8,9,21,22^.

Conversely, the experimental task used in the present study did not involve the detection of temporal or spatial errors, but rather involved the detection of regularity between an action and its outcome, which is a nonlinear disturbance. In a previous study^31^, the relationship between the two was examined by performing an action-outcome regularity detection task and a motor control task. In the motor control task, participants were asked to manipulate a 10% or 40% controlled dot for 10 s. The authors found no significant correlation between accuracy in the action-outcome regularity detection task and performance of the motor control task, indicating that the ability of participants to detect regularity was not related to how well they actually controlled an object^31^. On the basis of the previous and current results, there does not appear to be a significant relationship between perceptual sensitivity to action-outcome regularity and hand motor performance. In addition, the M-ABC2 manual dexterity test used in the current study is an assessment battery to identify children with significant motor skill deficits and may not have adequately captured the subtleties of sensorimotor functions in typically developing children.

However, the present results do not suggest that there is no relationship between the perceptual sensitivity of action-outcome regularity and motor performance in children. A previous study showed that there was an important relationship between the perception of action-outcome regularity and adaptive motor learning performance in adults^33^; in addition, a significant correlation was detected between the increase in SoA based on the perception of action-outcome regularity and the efficiency of adaptive motor learning^33^. Conversely, there was no significant correlation between the decrease in SoA based on the prediction error in the comparator model and the efficiency of adaptive motor learning^33^. Thus, there is a need for future studies to examine the relationship between the perceptual sensitivity of action-outcome regularity and motor function in children using adaptive motor learning tasks^26^ and online control tasks, such as the double-step reaching task^3–6,10^.

Between-group comparisons based on manual dexterity revealed that the perception of action-outcome regularity was significantly lower in children with low manual dexterity than in those with medium to high manual dexterity. Since there was no significant difference in age, sex, or preferred hand between the manual dexterity groups, manual dexterity was not influenced by these factors. Children with poor manual dexterity may have been unable to move their finger on the touchpad as they planned, resulting in difficulty in detecting the target dot. In future studies, recording finger movement trajectories and videos on the touchpad may allow a more detailed analysis of the relationship between manual dexterity and the perceptual sensitivity of action-outcome regularity. Children with a diagnosis of developmental coordination disorder (DCD) who score low on the M-ABC2 have dysfunction in the sensory-motor networks represented by the comparator model and mirror neuron system^55–60^. The present study revealed that children with low manual dexterity, but not as much as children with DCD, had a reduced ability to perceive action-outcome regularity. Therefore, children with DCD may not only have problems with motor control and motor learning systems but also with the perception of action-outcome regularity. Future studies examining the relationship between the perception of action-outcome regularity and impaired coordination of motor skills in children with DCD may help to improve our understanding of the pathogenesis of DCD and to develop rehabilitation techniques.

There were several limitations to the current study. The sample size and sex of each age-band group were not controlled. The age range examined in this study was 4–16 years. Therefore, future studies should be conducted with sample size and sex matched in each age band, and in a wide age range from children to the elderly to determine the developmental changes in the perception of action-outcome regularity. We hypothesized that the significantly lower detection of action-outcome regularity in 5–6-year-old children compared to 9–16-year-old children may be due to a local bias toward visual stimuli and the underdevelopment of visual attentional control functions in the younger children, but these functions were not measured. Therefore, future studies should clarify their relationship by measuring local–global preference, visual attentional control, and executive functions in conjunction with perceptual sensitivity to action-outcome regularity. In this study, the participants moved their preferred finger on the touchpad so that its movement was directly reflected in the movement of the target dot. However, the area of the touchpad dictated the spatial limits of free movements. Future studies should investigate how this differs from using a mouse, where spatial limits are more permissive. Especially in the younger participants who showed a local processing pattern for visual stimuli, the trajectory of their finger may have followed the trajectory of the 0% control dots set in motion by their finger movements. At this stage, there is no way to eliminate this possibility, but future studies may need to address it through measures such as improving the experimental task or using eye tracking.

## Conclusion

The present study examined the developmental changes in the perception of action-outcome regularity, which is involved in the generation of SoA. Our findings showed that the perceptual sensitivity of action-outcome regularity at 5–6 years of age was not at the same level as that at 9–16 years of age, and it was reduced in 5–16-year-old children with poor manual dexterity. Future studies should clarify the developmental changes in the perception of action-outcome regularity and take into account the limitations of the present study.

## Data Availability

The data that support the findings of this study are available from the corresponding author upon reasonable request.
